# Impact of cell type and species on RNA replication kinetics of Seoul virus

**DOI:** 10.1099/jgv.0.002189

**Published:** 2025-12-03

**Authors:** Autumn T. LaPointe, Stefan D. Klimaj, Alison M. Kell

**Affiliations:** 1 Department of Molecular Genetics and Microbiology, University of New Mexico School of Medicine, Albuquerque, NM, USA

**Keywords:** negative-stranded segmented virus, RNA replication, RT-qPCR, Seoul virus

## Abstract

Hantaviruses are zoonotic, tri-segmented, negative-sense RNA viruses and a significant public health threat. Viral pathogenesis varies between host species, with rodent reservoir infection being asymptomatic and human infection resulting in severe, immune-mediated disease. Viral pathogenesis is highly dependent on virus replication efficiency since it affects the virus’s ability to evade detection and determines the magnitude of the host immune response. However, the molecular replication kinetics for hantaviruses remain poorly defined. Therefore, we developed a sense- and segment-specific quantitative real-time PCR assay and an SYBR-based RT-qPCR assay, allowing us to quantify both negative-sense genome levels and total viral RNA synthesis of the small (S), medium (M), and large (L) segments of Seoul virus (SEOV). We then measured total viral RNA and genome accumulation in reservoir rat endothelial cells (RLMVEC), non-reservoir human endothelial cells (HUVEC-C), and Vero E6 epithelial cells. We also measured the ratio of each segment released into the culture supernatant, approximating the relative packaging efficiency. We found that, while the magnitude of viral RNA differed, RNA replication kinetics were largely similar between reservoir and non-reservoir endothelial cells. However, replication and release kinetics differed between infection of endothelial and Vero cells. We also found that the S, M, and L segments were not equally abundant during viral infection or release but instead followed a trend of M>L>S. Overall, this study validates two RT-qPCR assays to measure SEOV RNA, details the accumulation and release of each viral segment and demonstrates the impact of host cell type on hantavirus replication.

**Impact Statement**

Hantavirus infections in humans lead to significant immune-mediated morbidity and mortality around the world and pose a threat to public health. Zoonotic transmission occurs from persistently infected rodents which show no overt signs of disease. Understanding the molecular interactions that drive divergent infection outcomes is incomplete in the field of hantavirus research due to limited tools and reagents. This novel strand- and segment-specific RT-qPCR assay uncovers the unique kinetics of hantavirus genome replication in reservoir and non-reservoir cells. Further, this assay will serve as a valuable tool to investigate antiviral therapies and virus–host interactions critical for polymerase activity.

## Introduction

Hantaviruses are negative-sense, segmented RNA viruses which are maintained worldwide in rodent and insectivore reservoirs [1,2]. While infection of the rodent reservoir is typically asymptomatic and persistent, hantavirus infection of humans can result in severe disease [3]. In the case of Old World hantaviruses, disease manifests as haemorrhagic fever with renal syndrome with a mortality rate of up to 12% [4,5]. Meanwhile, infection with New World hantaviruses results in hantavirus cardiopulmonary syndrome with a mortality rate of up to 60% [6,7].

Hantavirus infection in both the rodent reservoir and human host primarily targets endothelial cells [8,9]. Infection of human endothelial cells leads to immune activation in cell culture, which may contribute to overall immune-mediated disease [7,10, 11]. For Old World hantaviruses, the RNA recognition receptors retinoic acid-inducible gene I (RIG-I) and melanoma differentiation-associated protein 5 (MDA5) are required for type I IFN signalling [7,11, 12]. The RNA pathogen-associated molecular pattern (PAMP) that is detected by RIG-I during hantavirus infection remains unknown. Hantavirus infection of the rodent reservoir is asymptomatic *in vivo* and fails to drive significant type I IFN signalling after viral entry *in vitro* [1,2, 13,15]. This lack of immune response is not due to direct antagonism of RIG-I by viral proteins [7]. It is therefore possible that the differences seen in immune response to Seoul virus (SEOV) infection between human and rat endothelial cells may be due to differential production of viral RNA PAMPs that could be detected by RIG-I. Alternatively, infection by incomplete viral particles that contain only one or two segments instead of three could also lead to immune sensing of improperly hidden PAMPs resulting in an inflammatory immune response. However, current knowledge of the RNA replication kinetics and genome release of SEOV in endothelial cells remains limited.

The SEOV genome is composed of three negative-sense RNA segments termed small (S), medium (M), and large (L) based on their respective sizes. During viral infection, two positive-sense RNA species are produced for each segment: the antigenome, which serves as a template for genome replication, and the viral mRNA [16,18]. Since the genomic RNA is packaged into viral particles, it represents the product of viral replication. As such, genome abundance represents the sum of viral replication and particle release. However, since we currently do not know which viral RNA species (i.e. positive-sense or negative-sense) leads to the activation of the immune response, understanding viral RNA synthesis as a whole is also important.

 In order to measure the RNA replication kinetics of SEOV, we developed a TaqMan strand-specific, quantitative, reverse-transcription, real-time PCR assay (ssRT-qPCR) to quantify viral genome abundance specifically, as well as a SYBR-based quantitative, reverse-transcription, real-time PCR (Sb-RT-qPCR) assay to measure total viral RNA synthesis for each segment. Using these assays, we measured SEOV RNA accumulation and release over time in cell cultures. Rat and human endothelial cells represent the target cells for SEOV infection, the reservoir and non-reservoir host, respectively. Additionally, we quantified SEOV RNA kinetics in Vero E6 cells, as they are an ubiquitous epithelial cell line used for hantavirus propagation and allowed us to measure SEOV replication in the absence of type I IFN. We hypothesized that there would be significant differences in RNA replication kinetics between the human and reservoir rat endothelial cells, but that RNA replication would be similar between the rat endothelial cells and Vero E6 cells, as neither mounts a significant type I IFN response to SEOV infection. However, we instead observed viral replication in both human and rat endothelial cells to have similar kinetics to each other, yet unique compared to infected Vero E6 epithelial cells. Similar to previous reports, overall RNA abundance in reservoir endothelial cells was higher than in human endothelial cells, suggesting a general infection advantage. We also noted differential abundance of the S, M, and L segments, following a consistent pattern of M>L>S across all three cell species. Collectively, this study presents a strand-specific RT-qPCR assay used to quantify SEOV RNA replication in reservoir and non-reservoir host species and suggests that cell type may impact viral replication kinetics.

## Methods

### *In vitro* transcription of SEOV RNAs

Full-length SEOV S genomic RNA was *in vitro* transcribed (IVT) from PCR templates using the T7 MEGAscript kit (Ambion AM1334) according to the manufacturer’s instructions. Full-length M and L genomes were unable to be obtained with this method, so 500 nt segments spanning the primer and probe binding regions were PCR-amplified using the primers in Table 1 (underlined portions indicate T7 promoter). These PCR templates were then IVT using the T7 MEGAshortscript kit (Ambion AM1354). For full-length antigenomic SEOV RNA, plasmids containing each of the positive-sense RNA segments with an upstream T7 promoter sequence were linearized with NheI and then used as templates for *in vitro* transcription using the T7 MEGAscript kit according to the manufacturer’s instructions.

**Table 1.** PCR primers for genomic IVT templates

S For	gcccgggtagtagtagctcccta
S T7 Rev	taatacgactcactatagggctagctagtagtatctcccta
M 500 nt For	ccacagctgcacttttgattac
M 500 nt T7 Rev	taatacgactcactatagggatttctgctgcacttgctg
L 500 nt For	ggttcttatttgtatcccagc
L 500 nt T7 Rev	taatacgactcactatagggcctataccggctgttgaaag

Underlined sequences indicate the T7 promoter.

To assess RNA integrity, RNA ladder (Thermo Scientific High Range RiboRuler FERSM1821) or IVT RNA was mixed with RNA loading dye containing ethidium bromide, heated to 80 °C for 10 min, placed immediately on ice and then run at 30 V on a 2% formaldehyde-agarose gel. RNA bands were visualized using a BioRad ChemiDoc imaging system. Concentrations of the RNA transcripts were measured using spectrophotometry, and the molecules of RNA per microliter for each transcript were calculated using the total molecular weight of each RNA segment.

### cDNA synthesis

IVT RNA was mixed with 50 µM dNTPs and either 1 µM (S and M) or 0.1 µM (L) cDNA primer (Table 2, uppercase indicates tag sequence), heated at 95 °C for 5 min and then immediately moved to ice. RT buffer (Maxima H-minus reverse transcription kit, Thermo Scientific K1652) and RNAse inhibitor (Abmgood G138) were then added to the reaction. The reaction was then moved to either 60 °C (M and L) or 65 °C (S), and Maxima H-minus reverse transcriptase was added. Reactions were then moved to 65 °C for 30 min, followed by 85 °C for 5 min and then cooled to 4 °C. RNase H (NEB M0297L) was added to each reaction and incubated at 37 °C for 20 min. To measure cDNA synthesis resulting from self-priming RNAs, cDNA synthesis was carried out in the absence of the cDNA primer. For both the validation assays using host RNAs and to experimentally measure intracellular viral RNA, 500 ng of cellular RNA was used as input. To measure genomic RNA release, 25% of RNA extracted from pelleted supernatants was used as input.

**Table 2.** cDNA primers

S genome	GCATGCTCGTGGACAGACTAaatcgtgactatatcagacaga
M genome	CTGCGTGAGTATCCTACCGCagaaccgattcaaagccattctacg
L genome	GCGCTACTGTGAGAGACGTAcaatctgcatgagcacatcc

Capitalized sequences correspond to PCR tag.

cDNA synthesis for total viral RNA Sb-RT-qPCR was done using a high-capacity cDNA reverse transcription kit (Applied Biosystems 4368814) following the manufacturer’s instructions. For both the validation assays using host RNAs and to measure intracellular viral RNA, 500 ng of cellular RNA was used as input. Validation assays using mixed host and IVT RNAs used 150 ng of cellular RNA as input.

### RT-qPCR

All primers and probes used were checked for self-complementarity and potential heterodimerization using ThermoFisher’s multiple primer analyzer and were also confirmed not to target either human (*Homo sapiens*) or rat (*Rattus norvegicus*) genes using NCBI’s Primer blast. The ssRT-qPCR assay was performed using 300 nM segment-specific qPCR primer, 300 nM segment-specific tag primer, 200 nM Taqman probe and PrimeTime Gene Expression Master Mix (IDT 1055771; Table 3). Standard curves were generated using cDNA made from IVT RNAs, such that a range of 1×10^6^ copies to 1 copy of cDNA was added to the ssRT-qPCR reaction. Thermocycling conditions for S were 95 °C for 3 min, 40 cycles of 95 °C for 15 s and 70 °C for 1 min. Thermocycling conditions for M and L were 95 °C for 3 min, 40 cycles of 95 °C for 15 s and 60 °C for 1 min. For infections, copy number per nanogram and copy number per milliliter were calculated using a standard curve from IVT genomic RNA.

**Table 3.** PCR primers and probes

S genome tag	GCATGCTCGTGGACAGACTA
S genome qPCR	cacctaattcagccatccctccg
S genome probe (Cy5)	tggctccatccctgcaagtgcacc
M genome tag	CTGCGTGAGTATCCTACCGC
M genome qPCR	cttctctgaatcggtgggtggc
M genome probe (Tamra)	acccactgtgaaccgacagaaactgc
L genome tag	GCGCTACTGTGAGAGACGTA
L genome qPCR	tcgctttgacgcatctcgt
L genome probe (Fam)	agggaattgatacagcacagccctca
S SYBR qPCR forward	gcaaattcatggcggagtct
S SYBR qPCR reverse	gttgcctgagggcttgaaat
M SYBR qPCR forward	tgttcaaggagagtgcccat
M SYBR qPCR reverse	gtgggtggcttgacaaactt
L SYBR qPCR forward	ttgagggctgtgctgtatca
L SYBR qPCR reverse	aaatccatcgctttgacgca

SYBR qPCR was performed using Applied Biosystems SYBR Green Universal master mix (4309155) following the manufacturer’s guidelines. cDNA was diluted to within the range of the standard curve and then mixed with 12.5 nM segment-specific forward and reverse primers and 1× SYBR mix. Thermocycling conditions were 95 °C for 10 min, 40 cycles of 95 °C for 15 s and 60 °C for 1 min. For infections, copy number per nanogram and copy number per milliliter were calculated using a standard curve from IVT genomic RNA.

### Cell culture and virus propagation

Vero E6 cells (ATCC, CRL-1586) were cultured in Dulbecco’s Modified Eagle’s Medium (DMEM) supplemented with 1% pen/strep, 1% nonessential amino acids, 10% heat-inactivated FBS and 2.5% HEPES. Primary rat lung microvascular endothelial cells (RLMVEC, VEC Technologies) were cultured in MCDB-131 base medium (Corning) supplemented with VascuLife VEGF LifeFactors (Lifeline Cell Technology LS-1020) and 10% heat-inactivated FBS. Human umbilical vein endothelial cells (HUVEC-C; ATCC, CRL-1730) were cultured in VascuLife EnGS Endothelial medium (Lifeline Cell Technology LM-0002) with VascuLife EnGS LifeFactors (Lifeline Cell Technology LS-1019) and 10% heat-inactivated FBS. All cells were cultured at 37 °C and 5% CO_2_. Endothelial cells were cultured on tissue culture-treated plates coated with rat tail collagen (VWR, 47747-218).

To propagate the virus, Vero E6 cells were infected with SEOV strain SR11 for 12 days [multiplicity of infection (MOI) of 0.01]. Supernatant was then collected and clarified by centrifugation at 1,000 ×***g*** for 10 min.

### Virus infections

RLMVEC, HUVEC and Vero E6 cells were infected with SEOV at a cell-specific MOI of 0.1 FFU per cell. Cell-specific MOI was determined previously [7]. After a 1 h adsorption period at 37 °C, the inoculum was removed, and the cells were washed twice with 1× PBS to remove unbound virus. Each cell line was given its respective media and incubated at 37 °C and 5% CO_2_. At the indicated times post-infection, supernatant was collected and clarified at 1,000 ×***g*** for 10 min. An aliquot (25%, 500 µL) of supernatant was kept for measuring viral titer, while the rest (75%, 1.5 mL) was inactivated by applying UV radiation at 104,000 µJ cm^−2^ (FisherBrand UV crosslinker, 13-245-221). Virus from UV-inactivated supernatant was pelleted through a 30% w/v sucrose cushion in TNE buffer (10 mM Tris-HCl pH 7.5, 100 mM NaCl, 1 mM EDTA) for 2 h at 30,000 rpm TRIzol reagent was added to the pellets, and total RNA was extracted via isolation following the manufacturer’s instructions. Cell monolayers were washed with 1× PBS and harvested in TRIzol reagent (Thermo Fisher Scientific, 15596026). Total RNA was extracted via isolation following the manufacturer’s instructions.

### Focus-forming unit assay

Standard virological FFU assays were used to determine the infectious titer of all viral samples as previously described [11]. Briefly, Vero E6 cells were inoculated with fivefold or tenfold serial dilutions of virus-containing samples. After a 1 h adsorption period, cells were overlaid with 2% methylcellulose supplemented with 2× DMEM, 2% FBS, 1% pen/strep and 1% HEPES. At 7 dpi, cells were fixed with 95% EtOH : 5% acetic acid and probed with primary anti-SEOV N (custom, Genscript) and HRP-conjugated goat anti-mouse antibodies (Jackson Immunoresearch, 115-035-003). Foci were visualized using the Vector VIP peroxidase substrate kit (Vector Laboratories, SK-4600).

## Results

### RT-qPCR assay design

**Fig. 1.**
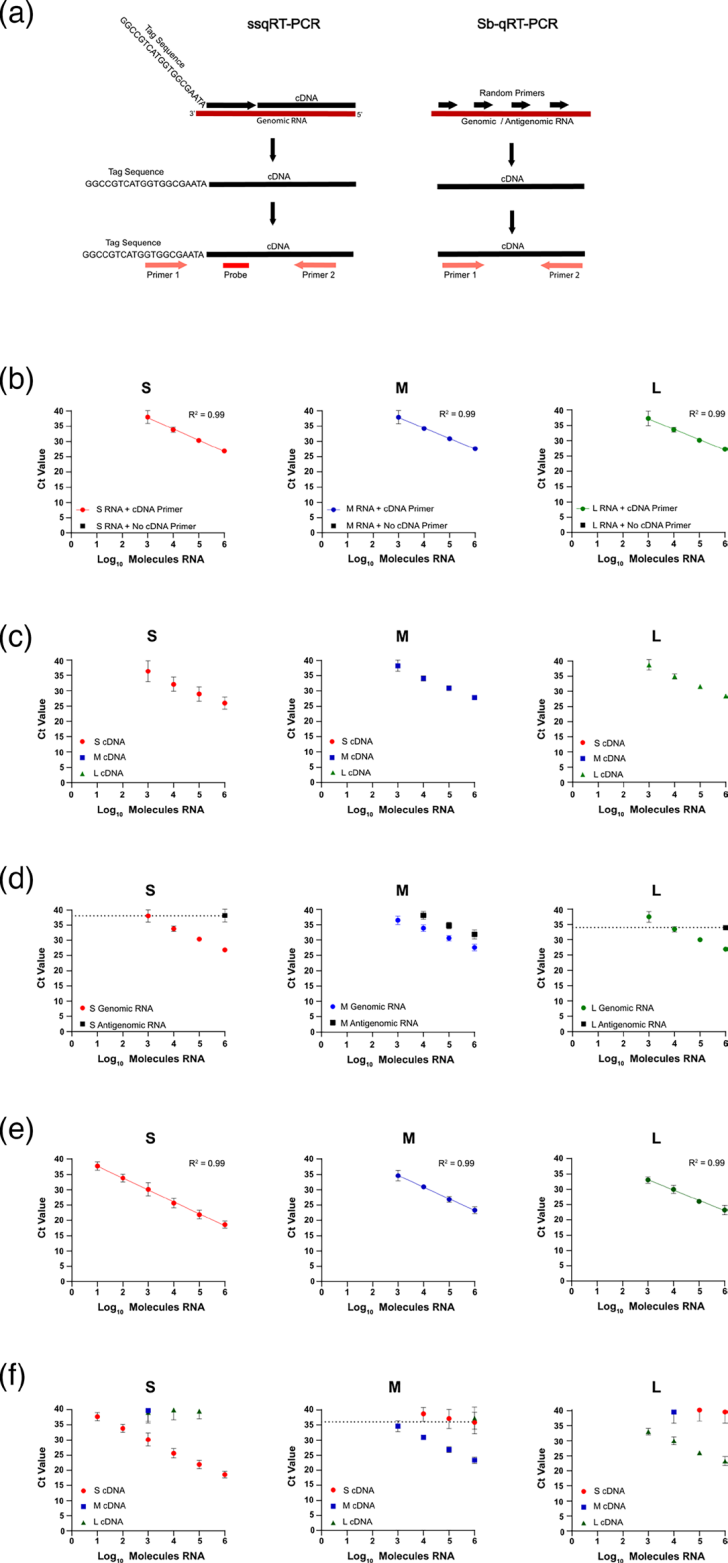
Validation of the ssRT-qPCR and Sb-RT-qPCR assays using IVT RNAs. (**a**) Schematic of the cDNA synthesis and qPCR strategies for the ssRT-qPCR and Sb-RT-qPCR assays. Standard curves using serial tenfold dilutions of either S, M, or L negative-sense IVT RNAs (red circles=S, blue squares=M, green triangles=L) in the ssRT-qPCR assays (**b–d**) or Sb-RT-qPCR assays (**e, f**). (**b, e**) Assay sensitivity was determined by using serial dilutions of S, M, or L IVT RNA with the paired primers/probe for each assay, and dotted lines represent C_t_ cutoff for reliable template quantification using the respective assay. (**c, f**) Segment specificity was determined by using S, M, or L IVT RNA individually against each segment’s primers/probe (primer/probe used is indicated in the graph titles). Data shown represent the mean of ≥3 independent experiments±sd.

The ssRT-qPCR was designed to be strand-, sense-, and segment-specific. To target the negative-sense genome for cDNA synthesis, we designed cDNA primers complementary to the genome for each segment. These primers included a unique tag sequence on the 5′ end to prevent self-priming (Fig. 1a) [19,20]. Real-time PCR primers targeting the unique tag sequence (3′) and viral genome (5′) were used to amplify the cDNA, and TaqMan probes specific for each segment were used for detection.

As a corollary to the ssRT-qPCR, we also designed segment-specific primers for a SYBR green assay (Sb-RT-qPCR) to measure total viral RNA (genome, antigenome, and viral mRNA) for each segment. As such, the Sb-RT-qPCR assay is only segment-specific as opposed to sense-specific. Random primers were used for cDNA synthesis so that both the negative- and positive-sense RNAs are intentionally primed. Segment-specific primers were then used for the subsequent RT-qPCR step (Fig. 1a).

### RT-qPCR assay validations

To validate our assays, we made negative-sense IVT RNAs for each SEOV segment from PCR templates. While we were able to transcribe the full-length S genome, we were unable to obtain full-length M and L genomes, so 500 nt segments spanning the primer and probe binding regions were used for the M and L genomes instead. These IVT RNAs were used as templates for first-strand cDNA synthesis. Utilizing first-strand reverse transcription minimizes any bias in efficiency due to differences in template length, as these efficiency differences are not repeatedly perpetuated in a single-cycle reaction as they would be in a typical, multi-cycle PCR reaction. The cDNA was then serially diluted to make a standard curve from 10^6^ to 10^0^ copies of input RNA for RT-qPCR. To confirm that the ssRT-qPCR assay detected only specifically primed RNA and not self-primed RNA, we included a negative control cDNA synthesis reaction without cDNA primers. For the S, M, and L negative-sense IVT RNAs, the ssRT-qPCR assay reliably detected down to 10^3^ molecules of input RNA, and when a line of best fit was applied, all three segments had R^2^ values of 0.99 indicating linear changes across the standard curve (Fig. 1b).

To test the segment specificity, IVT negative-sense RNAs of either S, M, or L were used as template for each segment’s assay (cDNA primer, PCR primers, and probe). For example, the S segment IVT RNA was used as template for the M assay primer/probe set, as well as for the L assay primer/probe set (Fig. 1c). For all three segments in the ssRT-qPCR assay, amplification was only detected when template RNA and primer/probe sequences were matched, demonstrating that the assays are segment-specific (Fig. 1c).

 To validate the sense specificity of the ssRT-qPCR assay, either genomic or antigenomic IVT RNA for each segment was used as the template for cDNA synthesis. For the S and L genomic primers and probes, antigenomic RNA was only detected when 10^6^ copies were added to the reaction, giving C_t_ values of 38 and 34, respectively (Fig. 1d). This correlates with an 11 C_t_ value difference in detection between the genomic and antigenomic RNAs for the S segment and a 7 C_t_ value difference in detection for the L segment. To address the potential contribution of antigenomic RNA to detection, C_t_ cutoffs were set at C_t_ 38 for S and C_t_ 34 for L, so that values falling at or below these cutoffs would be considered background detection for future experiments. Unlike S and L, M antigenomic RNA could be detected down to 10^4^ copies with the primers intended to detect only genomic RNA and only illustrated a 4.5 C_t_ value difference in detection between the genomic and antigenomic RNAs (Fig. 1d). Detection of the antigenomic M RNA remained consistent despite testing multiple different cDNA and PCR primers and PCR conditions (data not shown). Applying this cutoff for the M segment based on antigenome detection severely reduced the range of detection for the genome. Therefore, the primers and probe used for the M segment are not considered to be sense-specific. This validation test was only conducted for the ssRT-qPCR, where specificity for the negative-sense RNA was critical to the assay’s function, and not for the Sb-RT-qPCR assay, where both positive- and negative-sense RNA will be amplified and detected simultaneously.

For the Sb-RT-qPCR assay, the S segment was sensitive to 10^1^ molecules of input RNA, while the M and L segments were sensitive to 10^3^ molecules of RNA (Fig. 1e). When a line of best fit was applied to the standard curves, all segments had R^2^ values of 0.99. Sb-RT-qPCR segment specificity was tested the same as the ssRT-qPCR assay, where IVT negative-sense RNAs of either S, M, or L were used as the template for each segment’s assay (cDNA primer and PCR primers). For the Sb-RT-qPCR assay for S and L, non-target segments (M and L for S assay, S and M for the L assay) were detected at C_t_ values of 39 or greater, at or near the C_t_ cutoff of the assay (C_t_ 40) (Fig. 1f). In addition to this, the gap in detection between the intended segment and the non-target segments was ~9–10 C_t_ (equivalent to ~3 log). Given that the non-target segments were measured close to the C_t_ 40 cutoff of the assay and the large difference in C_t_ between the target and non-target segments, it was determined that detection of non-target segments was unlikely to significantly contribute to the measurement of the intended segment for S and L. The Sb-RT-qPCR assay for M detected non-target S and L segments at C_t_ values of ~36 when 10^6^ copies were added. To address the potential contribution of S and L RNA to the detection of M, a C_t_ cutoff was set at C_t_ 36 for M, so that values falling at or below this cutoff would be considered background detection.

 In order to compare SEOV replication kinetics during infection of reservoir and non-reservoir species, it was important to validate that the primers and probes designed for the ssRT-qPCR and Sb-RT-qPCR assays did not detect cellular RNAs from either the rodent reservoir or human cells. To test this, 500 ng of cellular RNA isolated from uninfected human umbilical vascular endothelial cells (HUVEC-C) or primary rat lung microvascular endothelial cells (RLMVEC) was used as the template for cDNA synthesis followed by either ssRT-qPCR or Sb-RT-qPCR (Fig. S1, available in the online Supplementary Material). None of the ssRT-qPCR assays detected host RNAs from either HUVEC or RLMVEC (Fig. S1A). For the M segment Sb-RT-qPCR, host RNAs were detected at 39 C_t_, but these were greater than the previously set C_t_ cutoff and would be treated as background in future experiments (Fig. S1B).

**Fig. 2.**
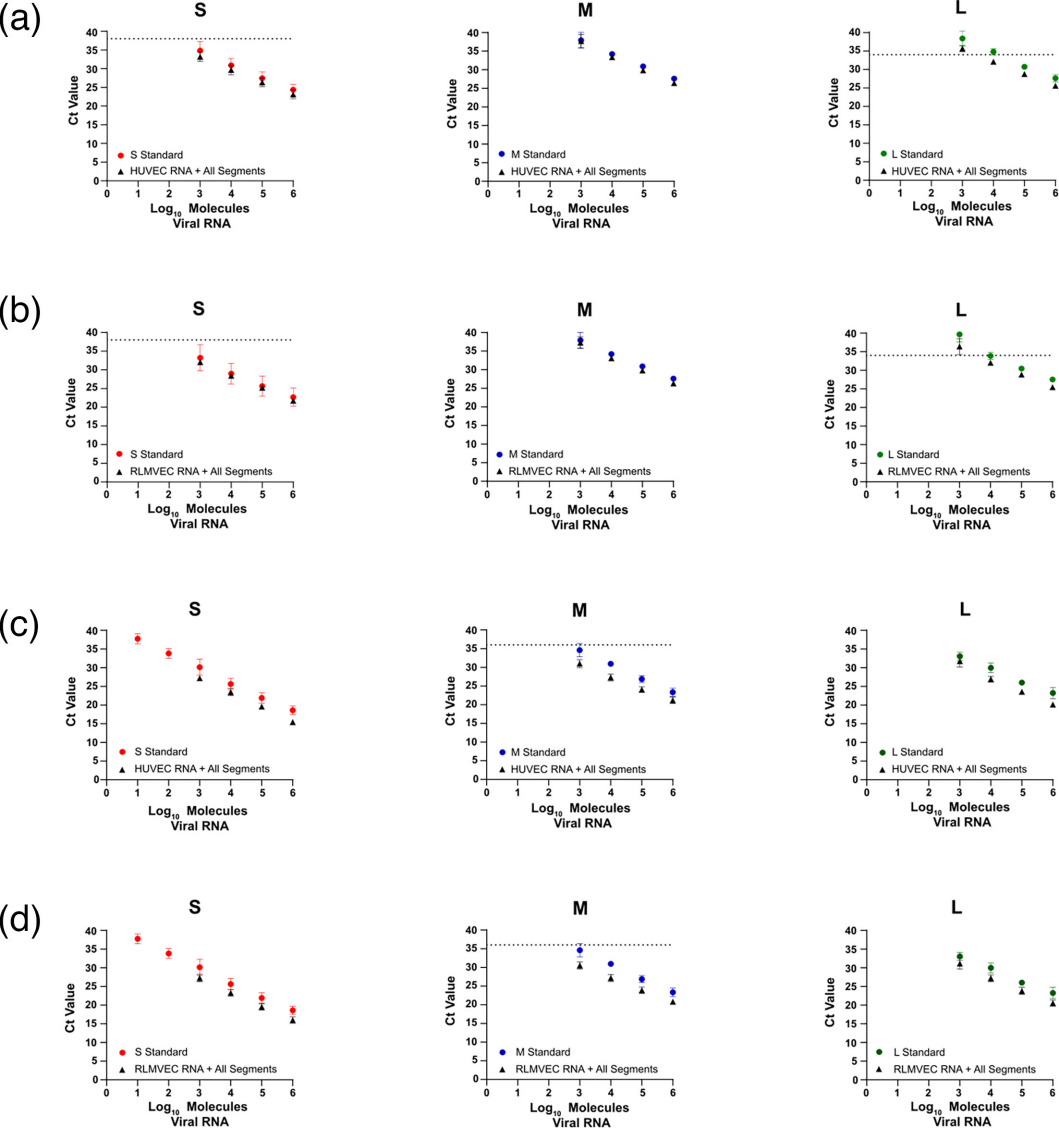
ssRT-qPCR and Sb-qPCR assays do not detect RNAs from uninfected host cells. To validate that neither the ssRT-qPCR assay (**a, b**) nor the Sb-RT-qPCR assay (**c, d**) detected host cellular RNAs, standard curves were made using serial dilutions of S, M, or L negative-sense IVT RNA (circles) or a mixture of 10^6^ copies per strand of S, M, and L genomic and antigenomic IVT RNAs plus 150 ng of either uninfected HUVEC (**a, c**) or uninfected RLMVEC (**b, d**) RNA (triangles). The IVT RNA alone or a mixture of IVT RNAs and uninfected cellular RNA was then used as input for either the ssRT-qPCR assay (**a, b**) or Sb-RT-qPCR assay (**c, d**) using the primers/probe indicated in the graph title. Data shown represent the mean of ≥3 independent experiments±sd.

We further demonstrate that the addition of non-target RNAs did not interfere with the detection of the intended viral RNAs. To do this, 150 ng of either HUVEC or RLMVEC RNA was mixed with 10^6^ copies of each of the genomic and antigenomic S, M, and L IVT RNAs. This mixture was then used as a template for either the ssRT-qPCR or Sb-RT-qPCR assay and was compared to a standard curve for the target genome or segment. For both the ssRT-qPCR assay (Fig. 2a, b) and Sb-RT-qPCR (Fig. 2c, d), the dilution series made by the host/virus RNA mixture closely mirrored the standard curves made using a single genomic IVT RNA for all three segments, indicating that the non-target cellular or viral RNAs do not interfere with target RNA detection for either assay.

### Quantification of viral RNA in SEOV infection

**Fig. 3.**
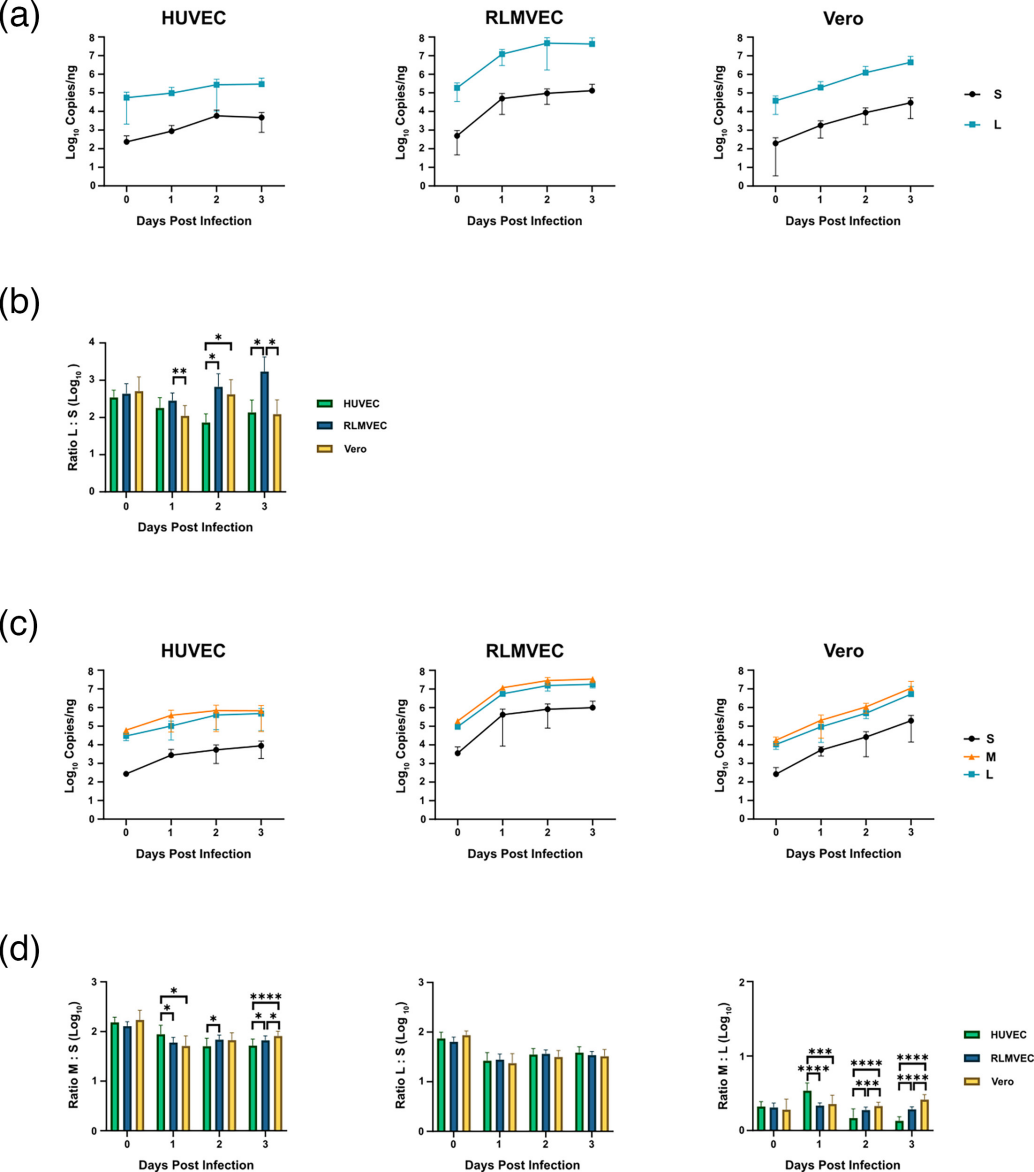
Quantification of viral RNA in SEOV-infected reservoir and non-reservoir cells. HUVEC, RLMVEC, and Vero cells were infected with SEOV at an MOI of 0.1 FFU per cell. Cells were harvested, and RNA was extracted at the indicated days post-infection. (**a**) S and L genomic RNA accumulation was measured using ssRT-qPCR for each cell type. (**b**) The ratio of L RNA copies to S RNA copies was then calculated for each time point. (**c**) Combined positive- and negative-sense viral RNA accumulation was measured using Sb-RT-qPCR for all three segments. (**d**) The ratios of M RNA copies to S RNA copies, L RNA copies to S RNA copies, and M RNA copies to L RNA copies were then calculated for each time point. Data shown represent the mean of ≥3 independent experiments±sd. Statistical significance determined by Student’s t-test. *, *P*<0.05; **, *P*<0.01; ***, *P*<0.001; ****, *P*<0.0001.

To measure SEOV RNA replication kinetics, HUVEC, RLMVEC, and Vero E6 cells were infected with a cell-specific MOI of 0.1 FFU per cell. Cell-specific MOI was determined as previously described to ensure that each cell type received the infectious units required for 10% infection of the culture [7]. This was done to account for differences in cell susceptibility to infection, allowing us to study RNA accumulation from a more equivalent baseline of infection. Cells were harvested every 24 h over 3 days to capture viral RNA accumulation. Because the ssRT-qPCR assay to detect the M genome was not sense-specific, only the S and L genomes were quantified in SEOV-infected cells. Viral replication in HUVEC displayed a ~3.7-fold increase in S genomic RNA from 0 dpi to 1 dpi and a ~6.7-fold increase in S genomic RNA, while the L genomic RNA increased ~1.7-fold from 0 dpi to 1 dpi and increased ~2.8-fold from 1 dpi to 2 dpi (Fig. 3a, Table S1). After 2 dpi, both S and L genomic abundance in HUVEC plateaued. S and L genomic RNA levels in infected RLMVEC increased ~101-fold for S and ~65-fold for L from 0 dpi to 1 dpi but then remained largely the same for the remainder of the infection. Conversely, infection of Vero cells resulted in increases in S and L genomic RNA across all 3 days. The S genome levels in infected Vero cells increased ~9-fold from 0 dpi to 1 dpi, ~5-fold from 1 dpi to 2 dpi, and ~3.5-fold from 2 dpi to 3 dpi. Meanwhile, L genome levels increased ~5-fold from 0 dpi to 1 dpi, ~6-fold from 1 dpi to 2 dpi, and ~3.5-fold from 2 dpi to 3 dpi. Cumulatively, from 1 dpi to 3 dpi, the S genome levels increased ~16.4-fold, and the L genome increased ~22.4-fold in infected Vero cells.

The ratio of L genome to S genome produced was also calculated for each cell type over the course of infection. For all three cell cultures, the L genome was found to be more abundant than the S genome at all time points. In general, the significantly higher ratio of L : S produced during infection of RLMVEC means a greater abundance of L genome was produced relative to S genome compared to the HUVEC or Vero cells (Fig. 3b, Table S2). Additionally, the ratio of L : S in RLMVEC increased over the course of infection, while the ratio of L : S remained more equivalent across the 3 days for infected HUVEC and Vero cells.

We also used the Sb-RT-qPCR assay to measure the combined accumulation of the genome, antigenome, and mRNA for each segment. This allowed us to quantify viral RNA synthesis as a whole as opposed to viral replication of the genome alone. Again, we observed that, after an initial increase in abundance between 0 dpi and 1 dpi, the abundance for all three segments remained steady in HUVEC and RLMVEC (Fig. 3c, Table S3). S, M, and L RNA levels increased ~12-fold, ~6.3-fold, and ~3.4-fold, respectively, from 0 dpi to 1 dpi in infected HUVEC but then only increased ~2.4-fold, ~1.7-fold, and ~4.6-fold, respectively, between 1 dpi and 3 dpi in infected HUVEC. Meanwhile, in infected RLMVEC, S, M, and L RNA levels increased ~117-fold, ~61.6-fold, and ~58.6-fold, respectively, from 0 dpi to 1 dpi, but then increased only ~2.1-fold, ~3-fold, and ~3.1-fold, respectively, from 1 dpi to 3 dpi. Consistent increases in abundance for all three segments were observed during Vero cell infection. S, M, and L RNA levels increased ~19.6-, ~11.7-, and ~8.8-fold, respectively, from 0 dpi to 1 dpi. All three segments increased ~5-fold from 1 dpi to 2 dpi, and both M and L RNA levels increased ~10-fold from 2 dpi to 3 dpi, while S RNA levels increased ~7.5-fold from 2 dpi to 3 dpi. Cumulatively, from 1 dpi to 3 dpi, RNA levels increased ~40-fold, ~50-fold, and ~56-fold for S, M, and L, respectively, in the infected Veros. The M segment was the most abundant in all three host species, followed by the L segment and then the S segment. Calculating the ratio of M : S for each cell culture found largely similar relative abundances (Fig. 3d, Table S4). Likewise, the ratio of L : S and M : L was also remarkably similar between cell cultures and over the course of infection. Although some of these ratios were found to be statistically significantly different between cell types, the magnitude of the difference is modest. Overall, while the RNA accumulation for each segment suggests that SEOV RNA replication may differ between these cells, the ratios between segments remain largely unchanged over the course of infection and between cell types.

**Fig. 4.**
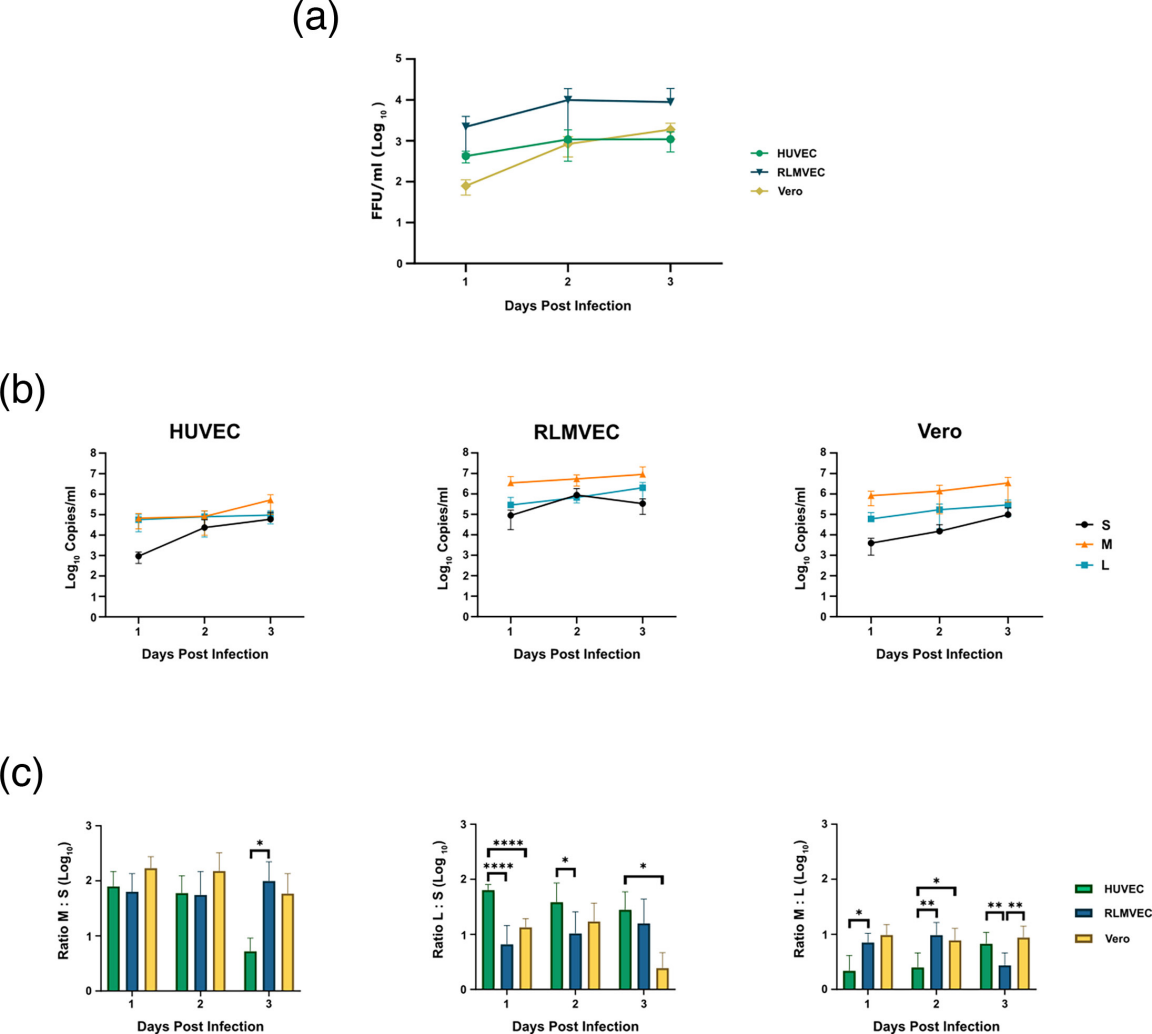
Quantification of infectious virus and RNA genome release from SEOV-infected cells. HUVEC, RLMVEC, and Vero cells were infected with SEOV at an MOI of 0.1 FFU per cell. (**a, b**) Supernatants were collected at the indicated times post-infection and used for either measuring viral titer via FFU assay (**a**) or RNA extracted to measure genomic RNA via the ssRT-qPCR assay (**b**). (**c**) Ratios of M RNA copies to S RNA copies, L RNA copies to S RNA copies, and M RNA copies to L RNA copies were calculated for each time point. Data shown represent the mean of ≥3 independent experiments±sd. Statistical significance determined by Student’s t-test. *, *P*<0.05; **, *P*<0.01; ***, *P*<0.001; ****, *P*<0.0001.

To determine whether the trends observed in genomic and total SEOV RNA synthesis were also observed in particle production, supernatants from the above infected cells were collected. An FFU assay was used to determine viral titer, while the ssRT-qPCR assay was used to measure genome release. Trends in viral titer largely follow what was observed for viral RNA replication, with titers from the human and rat endothelial cells increasing 2.5- and 4.5-fold, respectively, from 1 dpi to 2 dpi and then plateauing (Fig. 4a). Titer from Vero cells increased ~10.6-fold from 1 dpi to 2 dpi and then ~2.3-fold from 2 dpi to 3 dpi. To measure genome copies released from infected cells, viral particles were purified by ultracentrifugation through a 30% sucrose cushion to remove cell debris and naked RNA that might influence the ssRT-qPCR assay. Although the ssRT-qPCR was not used for measuring intracellular M genome in Fig. 3, this assay was used to measure the amount of M genome being released, as the negative-sense genome is the dominant RNA species in bunyavirus particles [21]. Importantly, this assay does not determine which segments are packaged together in a single virion, but simply the rate at which each segment is released in particles from the cell. The quantified abundance of each segment released largely reflected our intracellular observations, with M being the most abundant, followed by L and then S (Fig. 4b). S segment abundance increased during infection of HUVEC between days 1 and 2 post-infection ~25-fold and then plateaued, while M segment release remained equivalent between 1 dpi and 2 dpi and then increased ~6.5-fold from 2 dpi to 3 dpi (Table S5). L segment abundance remained steady across all 3 days. For infected RLMVEC, S segment abundance increased ~10-fold from 1 dpi to 2 dpi, then decreased ~2.5-fold from 2 dpi to 3 dpi. Meanwhile, the M segment remained steady over all 3 days, and the L segment only increased ~2.8-fold between 1 dpi and 2 dpi. S genome release from infected Vero cells increased consistently across all 3 days, ~4-fold from 1 dpi to 2 dpi and ~6.3-fold from 2 dpi to 3 dpi. M genome release increased ~2.6-fold from 2 dpi to 3 dpi, and L genome increased ~2.8-fold from 1 dpi to 2 dpi and then remained steady. Calculating the ratio of each segment released, the ratio of M : L segment released from HUVEC was significantly smaller compared to the ratio of M : L released from RLMVEC and Vero cells on days 1 and 2 post-infection (Fig. 4c, Table S6). Infected HUVEC also released a significantly larger ratio of L : S. Meanwhile, the ratios of M : S, L : S, and M : L released from infected RLMVEC and Vero cells are similar between the two cell types, with the exception of M : L at 3 dpi, where the ratio was significantly smaller for RLMVEC infection compared to Vero.

## Discussion

The strand-specific RT-qPCR assay can specifically measure the negative-sense genome segments of SEOV and represents the first system to do so for a hantavirus. To complement this assay and obtain a holistic view of SEOV replication, we also designed a SYBR-based RT-qPCR assay that measures the total viral RNA produced for each segment. The ssRT-qPCR and Sb-RT-qPCR assays allowed us to conduct an analysis of SEOV RNA replication and release in multiple cell types. This work builds on previous studies in the hantavirus field which used RT-qPCR to characterize the production of viral mRNA in Sin Nombre virus (SNV) and total viral RNA synthesis and release in Puumala virus (PUUV) [18,22]. While our study of SEOV total RNA replication indicated that the M segment was the most abundant in cells, followed by L and then S, SNV and PUUV were reported to produce the S segment most abundantly, followed by M and then L. The MOI used in these studies could be a technical explanation for this discrepancy. The previous studies used an MOI of 6 or 10 infectious units per cell in order to quantify viral RNA replication within the first hours of infection. Conversely, we conducted infections at an MOI of 0.1 FFU per cell to limit the potential impact of defective interfering particles. Using a higher MOI, while potentially necessary to measure RNA replication at early time points, could lead to multiple simultaneous entry events and replication of defective genomes [23,25]. This could result in an increased abundance of a particular segment that would not occur if only one infectious unit entered a cell. An additional biological explanation for the differences we see in segment abundances is that SNV and PUUV genomes encode an additional nonstructural protein on their S segment, termed NSs, not found in SEOV [26,27]. While NSs expression or putative sequences have been observed for hantaviruses with rodent reservoirs in the Cricetidae family (e.g. SNV, Andes virus, and PUUV), hantaviruses that infect rodents from the Muridae family (e.g. SEOV and Hantaan virus) lack an NSs [28,29]. Since both SNV and PUUV encode both the NSs and N on the S segment, it is possible that hantaviruses with an NSs might produce more S segment than those which only encode one protein on the S segment. A more extensive study measuring the RNA replication of multiple hantaviruses at different MOIs and times post-infection is necessary to clarify the relative differences seen in segment abundance between these studies. Interestingly, a recent study involving distantly related phleboviruses found the M genome UTRs of Heartland virus to be the most efficient for transcription and replication, followed by the L UTR and then the S UTR [30]. Future work applying the SEOV minigenome system could determine whether this is also true for hantaviruses and might explain our findings here [31]. Furthermore, while the current study focused on utilizing the ssRT-qPCR and Sb-RT-qPCR assays to measure SEOV, they have not been validated as specific for SEOV compared to other hantaviruses and therefore should not be used in a diagnostic setting. Determining the viral specificity of these assays remains an avenue for future work.

Notably, using the SYBR assay, the ratio of total RNA species quantified between segments remained fairly similar over the course of infection, especially the ratio of L : S. This suggests that total RNA of each segment is produced at similar rates. The fact that the ratios of the total RNA produced for each segment are largely consistent during infection means that the differences seen in RNA copy abundance between segments are not due to the rates at which each segment is produced but instead are likely due to early infection events. One potential explanation could be unequal packaging of S, M, and L genomes into particles in the viral stocks, which could lead to differences in RNA abundance after viral entry. However, for this to be the sole cause for the differences in abundance between each segment, there would need to be an almost two-log difference between incoming genome segments. Therefore, unequal packaging of genomic segments in the viral stock particles is unlikely to be the sole contributing factor. Alternatively, it is also possible that replication of the S, M, and L segments is initiated at different times, leading to the difference seen in RNA abundance. This idea that replication of each segment may start at different times is supported by Hutchinson *et al*., who observed that amplification of the S mRNA was the first to be detected at 4 hpi, followed by M mRNA at 8 hpi, and then L mRNA at 48 hpi in SNV infection [18]. Minor differences in segment ratios for total viral RNA synthesis over the course of infection of all three cell types suggest a conserved mechanism for regulating viral RNA production. Therefore, targeting these conserved mechanisms used for RNA replication or the pathways that dictate when replication of each segment is initiated may be useful strategies for antiviral development.

Similar to what has been observed for PUUV, general trends in genome release reflect total intracellular viral RNA [22]. We observed that in both genome release and intracellular RNA synthesis, M is the most abundant, followed by L and S. We also observed similar kinetic trends, with infected HUVEC and RLMVEC showing slight increases in genome release, but consistently larger increases in genome release as Vero infection progressed. However, one interesting difference observed was that the abundance of M and L genomes that were released from infected HUVEC is very similar compared to infection of the other two cell types, where M and L genome abundance differs by a log or more. Future studies will be needed to determine if these differences in SEOV genome release are solely immune-mediated or result from differences in host:virus interactions.

While it was unexpected to find that the segments were released in different ratios, it is important to note that the RT-qPCR assays used do not determine co-packaging or whether the RNA is replication competent, but only the abundance of each segment in the purified supernatant. It is possible that the abundance of replication-competent/infectious RNA for each segment is similar and that the increased abundance of M and L RNA in the supernatant is representative of increased defective RNA production for those segments. It is also possible that additional copies of M and L are needed to efficiently initiate viral replication in a newly infected cell. In a previous study by Bermúdez-Méndez *et al*. using the orthobunyavirus, Schmallenberg virus (SBV), the authors found relative segment abundance to be dependent on the cell line infected [32]. Specifically, Vero cells infected with SBV were found to release different abundances of the S, M, and L genomes, but infection of C6/36 mosquito cells resulted in the release of more equivalent amounts of all three segments. Therefore, it is possible that relative segment abundance may be context- and virus-dependent and warrants future study.

A further consideration is the possibility that purified supernatants contain viral particles along with extracellular vesicles. Endothelial cells have been reported to generate extracellular vesicles for communication with other cells, and viral RNA has been found within extracellular vesicles in other viral systems. Thus, the composition of particles in the cellular supernatant may influence the measured abundance of each segment and may be found to play a role in infection dynamics and pathogenesis for hantaviruses in the future.

Contrary to our hypothesis, although SEOV RNA was more abundant in the rat endothelial cells compared to human endothelial cells, a finding consistent with previous literature, the temporal kinetics of RNA accumulation and release remained similar between the two species [33]. The human and rat endothelial cells reach maximum RNA accumulation and viral titer by 2 dpi. In contrast, the Vero cells show consistent increases throughout infection. These data suggest that SEOV may replicate differently in endothelial cells versus epithelial cells. However, it is important to note that infection of Vero cells, while epithelial, is not representative of what infection of reservoir or non-reservoir epithelial cells is, as Vero cells lack a type I IFN response. Therefore, analysis in additional relevant epithelial cell types and additional strains of SEOV would be of interest. The lack of a strong type I IFN response during SEOV infection of RLMVEC and Vero cells suggests that these temporal differences are not driven solely by innate immune responses but rather may be cell-type specific [7]. Additionally, SEOV has been proposed to use the same receptors, αVβ3 integrins, on both Vero and endothelial cells, indicating that receptor usage may not explain this difference [34]. Therefore, it is possible that the variance seen in replication kinetics between the endothelial cells and the epithelial cells is due to the unique virus:host interactions that occur within each cell type. As such, future work will focus on defining virus:host interactions unique to reservoir and non-reservoir infections and their consequences for SEOV replication.

## Supplementary material

10.1099/jgv.0.002189 Uncited Fig. S1.

## Abbreviations

DMEM: Dulbecco’s Modified Eagle’s Medium
dpi: days post infection
FFU: focus forming unit
HUVEC-C: human umbilical vein endothelial cells
IFN: interferon
IVT: *in vitro* transcribed
L: large
M: medium
MOI: multiplicity of infection
PAMP: pathogen-associated molecular pattern
PUUV: Puumala virus
RIG-I: retinoic acid-inducible gene I
RLMVEC: rat lung microvascular endothelial cell
RT-qPCR: quantitative real-time PCR
S: small
Sb-RT-qPCR: SYBR-based quantitative, reverse-transcription, real-time PCR
SBV: Schmallenberg virus
SEOV: Seoul virus
SNV: Sin Nombre virus
ssRT-qPCR: strand-specific, quantitative, reverse-transcription, real-time PCR assay
