# Supplementary material for: Impact of cell type and species on RNA replication kinetics of Seoul virus
Source: J Gen Virol. 2025 Dec 3;106(12):002189. doi: 10.1099/jgv.0.002189 (PMC12674595; doi:10.1099/jgv.0.002189)
Supplement: Uncited Fig. S1. [file jgv-106-02189-s001.pdf]

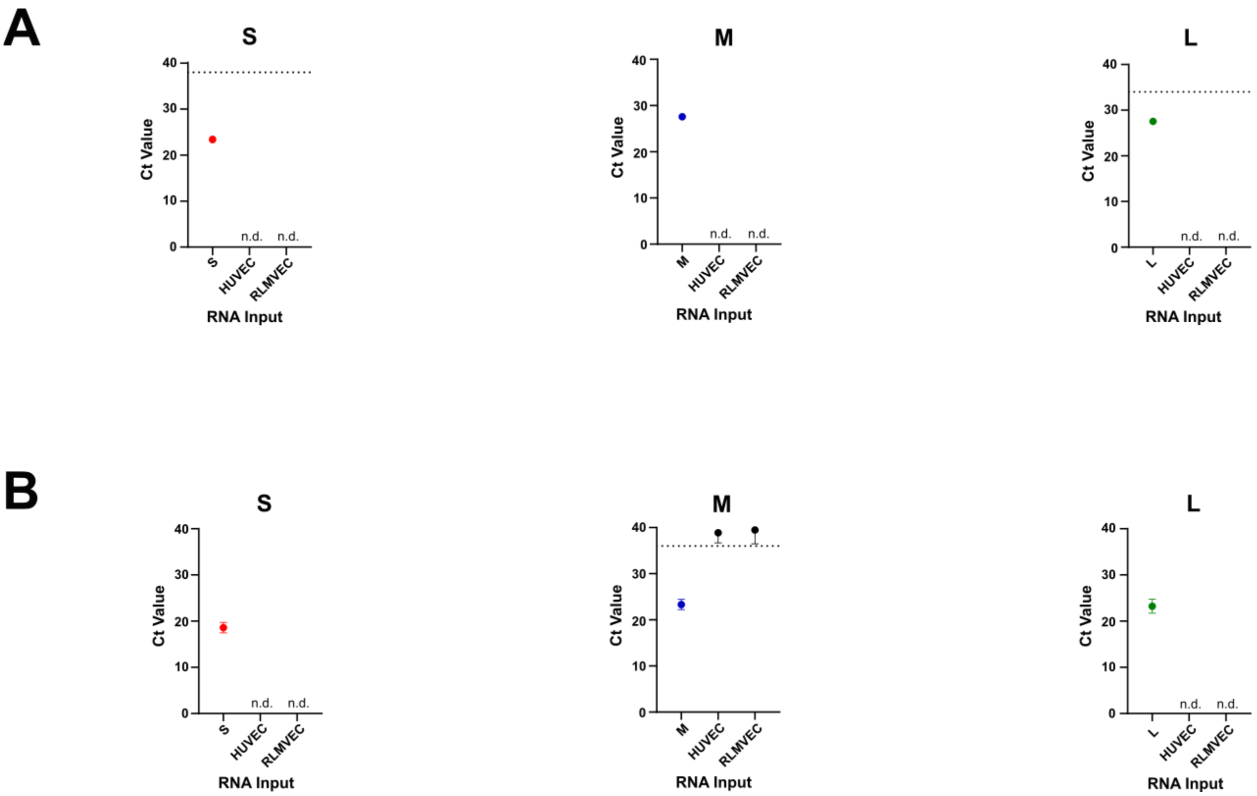

Supplemental Figure 1. ssqRT-PCR and Sb-qPCR assays do not detect RNAs from uninfected host cells. 500ng RNA isolated from uninfected HUVEC or RLMVEC was used as input for cDNA synthesis using the indicated segment primers (ssqRT-PCR; A) or random primers (Sb-qRT-PCR; B). Resulting cDNA was quantified using the respective ssqRT-PCR primers and probe or the SB-qRT-PCR primers. Data shown represent the mean of ≥3 independent experiments ±SD.

Supplementary Table 1. Fold Change of Genomic Viral RNA Over Course of Infection.

		0dpi -> 1dpi	1dpi -> 2dpi	2dpi -> 3dpi
HUVEC	S	3.73	6.71	0.81
	L	1.72	2.84	1.09
RLMVEC	S	101.54	1.88	1.40
	L	64.93	3.83	0.90
Vero	S	9.18	4.84	3.39
	L	5.09	6.32	3.56

Supplementary Table 2. Genomic RNA Segment Ratios.

		S : L
HUVEC	0dpi	1 : 343
	1dpi	1 : 180
	2dpi	1 : 73
	3dpi	1 : 136
RLMVEC	0dpi	1 : 433
	1dpi	1 : 282
	2dpi	1 : 667
	3dpi	1 : 1702
Vero	0dpi	1 : 509
	1dpi	1 : 110
	2dpi	1 : 417
	3dpi	1 : 122

Supplementary Table 3. Fold Change of Total Viral RNA Over Course of Infection.

		0dpi -> 1dpi	1dpi -> 2dpi	2dpi -> 3dpi
HUVEC	S	12.09	2.33	1.02
	M	6.34	1.80	0.97
	L	3.43	3.88	1.20
RLMVEC	S	117.16	1.96	1.21
	M	61.61	2.42	1.19
	L	58.60	2.78	1.15
Vero	S	19.64	5.06	7.44
	M	11.71	5.24	10.10
	L	8.84	5.42	10.46

Supplementary Table 4. Total Viral RNA Segment Ratios.

		S : M	S : L	L : M
HUVEC	0dpi	1 : 152	1 : 74	1 : 2.1
	1dpi	1 : 88	1 : 26	1 : 3.4
	2dpi	1 : 51	1 : 35	1 : 1.5
	3dpi	1 : 52	1 : 38	1 : 1.3
RLMVEC	0dpi	1 : 128	1 : 64	1 : 2.0
	1dpi	1 : 60	1 : 28	1 : 2.2
	2dpi	1 : 69	1 : 36	1 : 1.9
	3dpi	1 : 66	1 :34	1 : 1.9
Vero	0dpi	1 : 171	1 : 87	1 : 1.9
	1dpi	1 : 51	1 : 24	1 : 2.3
	2dpi	1 : 67	1 : 31	1 : 2.1
	3dpi	1 : 81	1 : 33	1 : 2.6

Supplementary Table 5. Fold Change of Genomic RNA from Viral Particles Over Course of Infection.

		1dpi -> 2dpi	2dpi -> 3dpi
HUVEC	S	24.88	2.53
	M	1.21	6.38
	L	1.38	1.21
RLMVEC	S	9.83	0.38
	M	1.58	1.65
	L	2.32	3.01
Vero	S	3.84	6.40
	M	1.66	2.53
	L	2.85	1.71

Supplementary Table 6. Particle Genomic Segment Ratios.

		S : M	S : L	L : M
HUVEC	1dpi	1 : 79	1 : 64	1 : 2.2
	2dpi	1 : 60	1 : 38	1 : 2.5
	3dpi	1 : 5.2	1 : 28	1 : 6.8
RLMVEC	1dpi	1 : 63	1 : 6.6	1 : 7.1
	2dpi	1 : 56	1 : 10	1 : 9.7
	3dpi	1 : 99	1 : 16	1 : 2.7
Vero	1dpi	1 : 170	1 : 13	1 : 9.7
	2dpi	1 : 151	1 : 17	1 : 7.8
	3dpi	1 : 59	1 : 2.4	1 : 8.7
